# Health state utilities associated with treatment for transfusion-dependent β-thalassemia

**DOI:** 10.1007/s10198-019-01136-0

**Published:** 2019-12-11

**Authors:** Louis S. Matza, L. Clark Paramore, Katie D. Stewart, Hayley Karn, Minesh Jobanputra, Andrew C. Dietz

**Affiliations:** 1 Patient-Centered Research, Evidera, 7101 Wisconsin Avenue, Suite 1400, Bethesda, MD 20814 USA; 2 bluebird bio, Cambridge, MA USA; 3 Patient-Centered Research, Evidera, London, UK

**Keywords:** Utility, Transfusion-dependent β-thalassemia, Stem cell transplant, Time trade-off, I10, I19

## Abstract

**Objectives:**

Transfusion-dependent β-thalassemia (TDT) is a genetic disease that affects production of red blood cells. Conventional treatment involves regular red blood cell transfusions and iron chelation, which has a substantial impact on quality of life. While potentially curative, allogeneic hematopoietic stem cell transplantation (allo-HSCT) is associated with risk of complications, including graft-versus-host disease (GvHD). Gene addition therapy, a novel treatment approach, involves autologous transplantation of the patient’s own genetically modified hematopoietic stem cells. The purpose of this study was to estimate utilities associated with treatment approaches for TDT.

**Methods:**

General population respondents in England valued eight health state vignettes (developed with clinician, patient, and parent input) in time trade-off interviews.

**Results:**

A total of 207 participants completed interviews (49.8% female; mean age = 43.2 years). Mean (SD) utilities for the pre-transplant health states were 0.73 (0.25) with oral chelation and 0.63 (0.32) with subcutaneous chelation. Mean utilities for the transplant year were 0.62 (0.35) for gene addition therapy, 0.47 (0.39) for allo-HSCT, and 0.39 (0.39) for allo-HSCT with acute GvHD. Post-transplant utilities were 0.93 (0.15) for transfusion independent, 0.75 (0.25) for 60% transfusion reduction, and 0.51 (0.38) for chronic GvHD. Acute and chronic GvHD were associated with significant disutility (acute = − 0.09, *p* < 0.0001; chronic = − 0.42, *p* < 0.0001).

**Conclusions:**

Utilities followed expected patterns, with logical differences between treatment options for TDT and substantially greater utility for transfusion independence than for ongoing treatment involving transfusion and chelation. These utilities may be useful in cost-utility models estimating the value of treatments for TDT.

**Electronic supplementary material:**

The online version of this article (10.1007/s10198-019-01136-0) contains supplementary material, which is available to authorized users.

## Introduction

Transfusion-dependent β-thalassemia (TDT) is a severe genetic disease caused by mutations in the *HBB* gene that lead to impaired or absent β-globin production, which affects the production of healthy red blood cells [1]. The incidence and prevalence of β-thalassemia vary across geographic regions. It is a rare disease in most of Europe and the US, but it is more common in some areas of South Asia, the Middle East, North Africa, and Southern Europe [2–6]. Conventional treatment for TDT involves lifelong supportive care with regular blood transfusions that lead to unavoidable iron build up that can result in significant organ damage [7, 8]. Therefore, patients require continuous and rigorous monitoring of iron burden and must adhere to an iron chelation regimen. The only currently available therapy with the potential to correct the genetic deficiency is allogeneic hematopoietic stem cell transplant (allo-HSCT), which carries the risk of serious complications, including graft-versus-host disease (GvHD), graft failure, and death [9–12].

One novel treatment approach is gene addition therapy. This approach involves the addition of functional copies of the β-globin gene into the patient’s own hematopoietic stem cells (HSCs), ex vivo. These modified HSCs are then introduced into the patient following myeloablative conditioning [13, 14]. Gene addition therapy using autologous HSCs may offer an alternative to allo-HSCT for patients with TDT who do not have a suitably matched related donor [15, 16]. Gene addition therapy using autologous HSCs would allow these patients to avoid the risk of GvHD and would not require immunosuppression to prevent graft rejection [15, 17]. As new treatments such as gene addition therapy are developed for TDT, cost–utility analyses (CUAs) are needed to examine their value and inform resource allocation decisions [18–20]. Such studies require health state utility values to calculate quality-adjusted life years (QALYs). Utilities are values anchored to 0 (dead) and 1 (full health) that quantify the strength of preference for health states.

Although CUAs have been conducted and published for TDT treatments, few utilities representing TDT health states have been published, and the available utilities have limitations. For example, several studies have focused on utilities associated with various types of iron chelation therapy (e.g., differentiating between oral and subcutaneous chelation), but these studies did not aim to quantify the burden of ongoing blood transfusion and iron chelation [21–24]. Two of these publications present health state vignettes, which do not mention some key side effects of iron chelation such as gastrointestinal upset, constipation, or damage to liver and kidney functioning [21, 23]. These publications also appear to understate the burden of chronic transfusions by omitting details on blood tests required prior to transfusions, the duration of transfusions, and potential complications associated with anemia in the days before transfusions such as difficulty concentrating, irritability, decreased productivity at work/school, limited ability to exercise, and pain [21, 23]. One of the studies derived utilities based on perceptions of nurses (rather than patients or general population respondents), which is not generally considered to be a preferred method of utility estimation [22].

Two studies were identified that used the EQ-5D health-related quality of life instrument to derive utilities for patients with TDT, and utility scores were reported up to 0.87 [24, 25]. These scores may not reflect the considerable burden of TDT and its treatment, possibly because the five domains of the EQ-5D are not sensitive to the specific impact of ongoing transfusion and iron chelation. The EQ-5D domains assess mobility, self-care, usual activities, pain/discomfort, and anxiety/depression. One of the domains (usual activities) could be confounded by the standard management of TDT as it is, in essence, a usual activity for individuals living with TDT. It seems likely that this ongoing burdensome treatment process could affect quality of life in ways not covered by these five items. Therefore, it is possible that the EQ-5D would underestimate the impact of these treatments on quality of life and thus overestimate the utility of TDT.

There are studies estimating utilities of related concepts, but relevance to patients with TDT is unknown. For example, several studies have focused on utilities associated with transfusions or stem cell transplant in the context of diseases other than β-thalassemia [e.g., myelodysplastic syndromes (MDS)] [26–30]. One study has estimated utilities associated with GvHD, but in the context of leukemia rather than TDT [28].

In summary, there are few published utilities for TDT, and those that are available have notable limitations. Therefore, the purpose of this study was to estimate utilities associated with TDT and its treatment. Health state descriptions (often called health states or vignettes) were developed to represent TDT with ongoing blood transfusions and iron chelation therapy, as well as the period of time for which patients undergo HSCT. Health states were developed to represent the experience of both allo-HSCT and auto-HSCT. In addition, several post-transplant health states were evaluated.

## Methods

### Overview of study design

This study was designed to estimate utilities associated with treatment for TDT. Vignette-based methodology was selected for two reasons. First, the vignette approach is often considered the best or possibly the only available method to estimate the utility impact associated with the treatment process [31, 32]. In this case, the relevant treatment processes include the ongoing cycle of transfusion and chelation, as well as differences between conventional and investigational HSCT procedures [33]. While health technology assessment (HTA) authorities often prefer that generic preference-based measures such as the EQ-5D are used to generate utilities [34], generic instruments are not designed to be sensitive to treatment process variables. In contrast, vignette-based methods are useful for this purpose because health states can be designed to focus on treatment process attributes. Therefore, almost all studies estimating treatment process utilities use the vignette-based approach [31]. Second, for rare diseases such as TDT [35], it may not be feasible to have standardized preference-based instruments completed by a large enough sample of patients to represent a range of specific health states. In contrast, hypothetical health states can be drafted based on input from smaller samples of clinicians and patients, and then valued by members of the general population without requiring a large sample of patients.

Health state descriptions were drafted based on published literature, clinician interviews, patient/caregiver interviews, and a pilot study. Eight hypothetical health states were presented during the utility interviews: five chronic (i.e., unchanging over time) health states describing patients with TDT pre- or post-transplant, as well as three “path states”. Path states are vignettes that describe changes over time [36–38]. In this case, the path states describe a series of typical health-related events during the year in which the patient would undergo HSCT. Unlike previous studies using health state vignettes to estimate utilities associated with TDT [21, 23], one aim of the current study was to estimate utility associated with the ongoing burden of chronic transfusions. Therefore, health states included details on blood tests required prior to transfusions, the duration of transfusions, potential complications associated with anemia in the days before transfusions, and key side effects of iron chelation.

Utilities for these health states were then elicited in a time trade-off (TTO) task with a 10-year time horizon for the five chronic health states and a 1-year time horizon for the three path states. The in-person, individual interviews were conducted by trained interviewers with general population participants in March 2018 in three locations in England (Newcastle, London, Bristol). Participants were required to provide written informed consent before completing study procedures, and all procedures and materials were approved by an independent institutional review board (Ethical & Independent Review Services; Study Number 17166).

### Health state development

A targeted literature search was performed to support the health state content and inform development of questions to be asked in subsequent interviews. The literature search focused on patient experiences with TDT [39–41], transfusions [25, 42, 43], iron chelation [21, 23, 24], allo-HSCT [9, 12, 44, 45], gene therapy [13], and GvHD [45, 46].

Multiple rounds of telephone interviews were conducted with clinicians including four hematologists and one registered nurse specializing in thalassemia, all of whom had extensive experience treating patients with TDT. Three of the clinicians were based in the UK, one in the US, and one in France. Interviews were also conducted with one adult patient with TDT from the US and one adult caregiver for an adolescent patient with TDT in the UK.

Health states were developed through an iterative process with the physicians, nurses, patient, and caregiver. Each expert participated in up to three discussions so they could respond to multiple drafts of the health states as they developed. Initial questions focused on gathering information that should be included in the health states (e.g., patients’ typical experience with TDT, transfusions/chelation, the transplant process, and GvHD). Follow-up discussions focused on reviewing and editing health state drafts to ensure that the descriptions of the treatment processes and symptoms were clear and accurate representations of the typical patient experience.

The two “A” health states describe a typical patient with TDT who has never had HSCT (i.e., “pre-transplant”). These two health states have identical descriptions of TDT, including symptoms, impact, and blood transfusions every 3–4 weeks. The only difference between these two health states is the description of iron chelation. Health state A1 describes oral iron chelation (deferasirox) taken on a daily basis; the potential side effects to liver, kidney, and gastrointestinal functioning; and typical monitoring for patients receiving this treatment. Health state A2 describes subcutaneous iron chelation (desferoxamine) using a small infusion pump about 5 days each week for about 10 h. This health state includes description of potential side effects on vision and hearing, along with the typical monitoring associated with this treatment.

The three “B” health states describe a typical series of events during the transplant year. These health states include sections describing preparation for transplant, the transplant itself, the first month after transplant, and recovery after leaving the hospital. Health states B1 and B2 describe gene addition therapy with autologous HSCTs and allo-HSCT, respectively. The key differences between these health states are the immune system suppression required before and after allogeneic transplant (but not before or after autologous transplant), time to limit exposure to others after transplant (a month or 2 vs. 6 months), and time until return to work (4–6 months vs. 9–12 months). B3 is the same as the allogeneic transplant health state (B2), except for the addition of acute GvHD symptoms and treatment.

The three “C” health states describe possible post-transplant outcomes. C1 and C2 differ in the need for transfusion and chelation following a stem cell transplant. Health state C1 describes transfusion independence without anemia, regular transfusions, or iron chelation. Only annual follow-up visits are required for monitoring. Health state C2 describes a reduced need for transfusion following the transplant (i.e., 60% reduction). Compared to health state A1 (pre-transplant transfusions and oral chelation), health state C2 requires transfusions less often (every 6–8 weeks). Health state C2 was intended to represent the possibility of transfusion reduction (rather than full transfusion independence) with gene addition therapy administered via autologous stem cell transplant [47]. Health state C3 describes transfusion independence identical to health state C1, except for the inclusion of chronic GvHD.

For the purposes of the TTO valuation, the A and C health states are chronic states that remain stable over a 10-year period. The B health states are path states describing a series of events that occur over a 1-year period. Health states were presented to participants on individual cards with bullet point text describing each state. The path states included a timeline illustrating the sequence and timing of events during the year. Health states did not include the label “transfusion-dependent β-thalassemia”. The condition was described as “an inherited blood disease”.

Although efforts were made to shorten and clarify the health states as much as possible, some health states remained relatively long so that they could include the details that clinicians said were necessary to adequately represent the typical patient experience. Therefore, several strategies were used to help respondents understand and attend to all the information. For example, health states B1 to B3 were initially presented separately from the others to avoid overwhelming the respondents with too much information at one time. In addition, all health states were formatted in a series of sections with headers to help organize the information. Furthermore, the three transplant health states included timelines illustrating the series of events. Finally, interviewers asked the participants to explain their preferences to ensure that they understood and considered all aspects of the health states.

See Supplementary Appendix for full text of the health states used in the valuation task.

### Participants

Participants were recruited from the general population and were required to be at least 18 years of age; able to understand the utility assessment procedures; willing to provide written informed consent; and a current resident of the UK. No specific clinical characteristics were required because this study was intended to estimate utilities for CUAs for submissions to health technology assessment agencies, which often prefer utilities representing general population values [34, 48, 49]. Participants were recruited via local newspapers and online advertising. Participants were compensated £50 as reasonable reimbursement for time and travel expenses associated with study participation.

### Pilot study

To assess the clarity of the health states and utility assessment methodology, a pilot study was conducted in London with 20 general population participants (55.0% male; mean age 42.4 years; age range 21–73 years). The pilot began with two participants reviewing all eight health states (A1–C3) and ranking them in order of preference. However, participants struggled when comparing the chronic health states to the path health states. Therefore, a decision was made to present the health states in three groups for the remaining participants: the A health states (pre-transplant), the B health states (the transplant year), and the C health states (post-transplant). Participants ranked the chronic states and path states separately. After the introductory ranking task, the health states were valued in a TTO interview. The ranking and TTO tasks were feasible for all respondents, and most participants reported that the health state language and content was clear and comprehensible. Some participants suggested minor edits in formatting and word choice, and the health states were revised accordingly.

### Utility interview procedures and scoring

After finalizing the health states and methods based on the pilot study, health state utilities were elicited in a TTO valuation study in March 2018. In the utility interviews, participants were first asked to rank the health states in order of preference (from most preferable to least preferable). The ranking task was included so that respondents could familiarize themselves with health state content and think about the preferences prior to the TTO task. After completing the ranking, participants valued the chronic health states in a TTO task with a 10-year time horizon with 6-month trading increments. For each health state, participants were offered a choice between living 10 years in the health state being rated or a shorter duration in full health. Choices were presented in a booklet with one page illustrating each choice (i.e., life 1 vs. life 2) in an order that alternated between longer and shorter amounts of time in full health (10 years, 0, 9.5, 0.5, 9, 1, 8.5, 1.5, …). The instructions from the interviewer were phrased as follows: “Now I am going to ask you to make choices. On each page, I am going to ask which life you would prefer. You can choose Life 1, Life 2, or they can be equal. Life 1 means that you live a certain amount of time in full health, followed by dead. The amount of time in full health is different on different pages. Life 2 means that you live 10 years in the health state being rated, followed by dead”.

The path health states were valued following the same procedure, but with a 1-year time horizon and 1-month trading increments. For health states that the respondent perceived as better than dead, utility scores (*u*) were calculated based on the point of indecision as the number of years/months in full health (*x*) divided by the number of years/months in the health state being rated, yielding a utility score on a scale with the anchors of dead (0) and full health (1).

When participants indicated that a health state was worse than dead, the task and scoring procedures were altered as described in previous literature [50, 51]. Participants were offered a choice between dead (choice 1) and a 10-year (or 1-year for the path states) life span (choice 2) beginning with varying amounts of time in the health state being rated, followed by full health for the remainder of the life span. The resulting negative utility scores were calculated with a bounded scoring approach commonly used to avoid highly skewed distributions for negative utility scores (*u* = − *x*/*y*, where *x* is the number of years/months in full health, and *y* is the number of years/months in the total life span of choice 2, which was 10 years for the A and C health states and 1 year for the B health states).

To maintain data quality and minimize logical inconsistencies, interviewers were trained to identify illogical responses. For example, it would be illogical for health state B3 (with GvHD) to be ranked as preferable to health state B2 (otherwise identical health state without GvHD). When respondents provided these unexpected preferences in the ranking or TTO tasks, the interviewer would gently query the response by asking the respondent to explain their preferences. Interviewers were trained to avoid biasing any responses or suggesting that any response was correct or incorrect.

Three outcomes of these queries were possible. (1) Most frequently, respondents independently recognized an error while explaining their responses. In these situations, respondents often asked to revise one or more answers, and they were permitted to do so. (2) Some respondents provided justification for unusual responses that seemed potentially illogical. If respondents provided an explanation demonstrating that they understood the health states and intended to respond in this way, then the unusual responses were considered acceptable. For example, four participants preferred health state A2 over A1 and provided an explanation reflecting a good understanding of the task and health states. Because these responses reflect a true preference rather than an error, the resulting rankings and utilities are included in the data set. (3) Sometimes, it became clear that participants provided a response without truly understanding the task or the health states. When this happened, the interviewer attempted to clarify any misunderstanding. However, there were three participants who seemed unable to understand either the health state content or the assessment procedures after multiple explanations. These three interviews were discontinued. The three participants were thanked for their time and paid for their participation, but their data were not included in the data set.

### Statistical analysis procedures

Statistical analyses were completed using SAS (version 9.4). Continuous variables including utilities and differences between health state utilities are summarized in terms of means and standard deviations, and categorical variables are summarized as frequencies and percentages. Disutility for acute GvHD was calculated by subtracting the utility of the acute GvHD health state (B3) from the utility of the allogeneic transplant health state (B2). Disutility for chronic GvHD was calculated by subtracting the utility of the chronic GvHD health state (C3) from the transfusion independent health state (C1). Student’s *t* tests and ANOVA with Scheffe’s post hoc comparisons were conducted to compare utility scores between various subgroups (e.g., gender, age, geographic location). Paired *t* tests were conducted to test whether there were significant differences between pairs of related health states (e.g., oral vs. subcutaneous chelation for pre-transplant TDT).

## Results

### Sample description

A total of 250 potential participants were scheduled for interviews. Of these, 211 attended the scheduled interview. One participant was found to be ineligible after starting the interview due to visual impairment, which made it impossible to read the health state cards. Three of the 210 eligible participants had difficulty understanding the health states and/or TTO procedures and were therefore unable to provide valid TTO data. Thus, 207 valid TTO interviews were conducted (87 in Newcastle, 72 in London, and 48 in Bristol). Participants were 49.8% female, with a mean age of 42.5 years (Table 1). The majority of participants reported ethnicity as White (84.5%). The most commonly reported health conditions were depression (21.3%), anxiety (20.3%), arthritis (9.2%), and hypertension (9.7%). No participants reported having β-thalassemia, but two participants (1.0%) reported knowing someone diagnosed with β-thalassemia.

**Table 1:** Demographic characteristics

	Newcastle (*N* = 87)	London (*N* = 72)	Bristol (*N* = 48)	Total sample (*N* = 207)	*p* value^a^
Age (mean, SD)	42.6 (16.4)	45.0 (14.2)	41.9 (15.1)	43.2 (15.3)	0.48
Gender, *n* (%)					
Male	44 (50.6%)	39 (54.2%)	21 (43.8%)	104 (50.2%)	0.53
Female	43 (49.4%)	33 (45.8%)	27 (56.3%)	103 (49.8%)	
Ethnicity, *n* (%)					
White	85 (97.7%)	46 (63.9%)	44 (91.7%)	175 (84.5%)	< 0.01
Mixed	2 (2.3%)	3 (4.2%)	2 (4.2%)	7 (3.4%)	
Asian	0 (0.0%)	14 (19.4%)	2 (4.2%)	16 (7.7%)	
Black	0 (0.0%)	8 (11.1%)	0 (0.0%)	8 (3.9%)	
Other^b^	0 (0.0%)	1 (1.4%)	0 (0.0%)	1 (0.5%)	
Marital status, *n* (%)					
Single	44 (50.6%)	45 (62.5%)	30 (62.5%)	119 (57.5%)	0.23
Married/cohabitating/living with partner	43 (49.4%)	27 (37.5%)	18 (37.5%)	88 (42.5%)	
Employment status, *n* (%)					
Full-time work	34 (39.1%)	31 (43.1%)	14 (29.2%)	79 (38.2%)	0.56
Part-time work	24 (27.6%)	19 (26.4%)	18 (37.5%)	61 (29.5%)	
Other	29 (33.3%)	22 (30.6%)	16 (33.3%)	67 (32.4%)	
Education level, *n* (%)					
University degree	28 (32.2%)	41 (56.9%)	21 (43.8%)	90 (43.5%)	0.01
No university degree	59 (67.8%)	31 (43.1%)	27 (56.3%)	117 (56.5%)	
Has at least one dependent child (*n*, %)					
No	39 (44.8%)	37 (51.4%)	32 (66.7%)	108 (52.2%)	0.10
Yes	48 (55.2%)	34 (47.2%)	16 (33.3%)	98 (47.3%)	
Missing	0 (0.0%)	1 (1.4%)	0 (0.0%)	1 (0.5%)	
Has at least one dependent adult^c^ (*n*, %)					
No	78 (89.7%)	63 (87.5%)	44 (91.7%)	185 (89.4%)	0.41
Yes	9 (10.3%)	7 (9.7%)	4 (8.3%)	20 (9.7%)	
Missing	0 (0.0%)	2 (2.8%)	0 (0.0%)	2 (1.0%)	

^a^*p* values are based on ANOVAs for continuous variables and Chi-square analyses for categorical variables

^b^Other ethnic/racial background included: Iranian (*n* = 1)

^c^Based on the question “Are there any other people besides children who depend on you to care for them (for example, elderly or disabled relatives)?”

### Health state utilities

In the introductory ranking task, participants compared and ranked the three path states describing the year in which a transplant occurs (B1, B2, and B3). B1 (gene addition therapy) was always ranked as most preferable, and B3 (allogeneic transplant with acute GvHD) was always ranked as least preferable, with B2 (allogeneic transplant) in the middle. Next, participants ranked the five chronic health states with rankings ranging from 1 (most preferable health state) to 5 (least preferable). Of these five health states, C1 (transfusion independent) was always ranked as most preferable. Rankings of the other four health states varied, with mean rankings of: 2.31 for C2 (post-transplant 60% transfusion reduction), 3.14 for A1 (TDT with oral chelation), 4.08 for A2 (TDT with subcutaneous chelation), and 4.47 for C3 (post-transplant chronic GvHD).

**Fig. 1:**
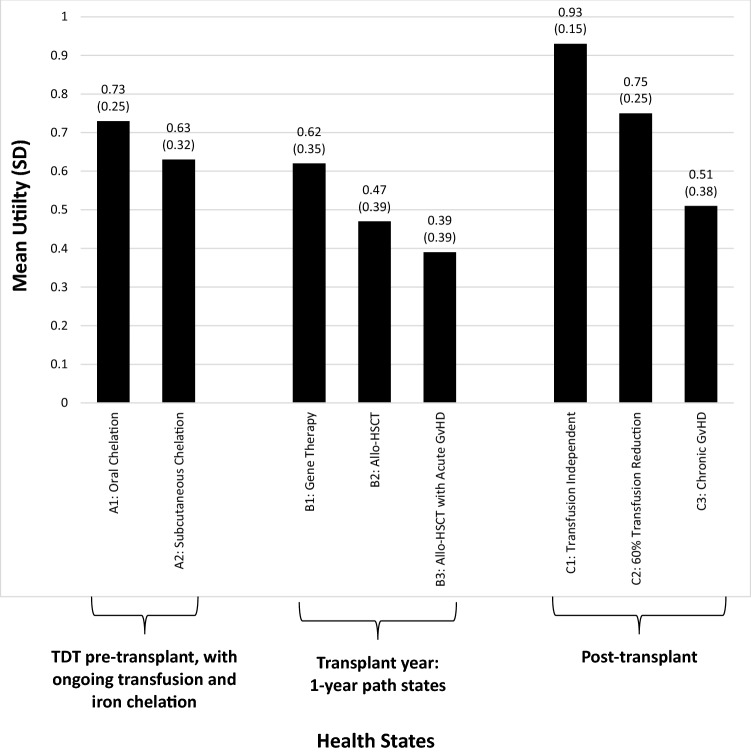
Mean health state utilities

Mean TTO utility scores are presented in Fig. 1. The pre-transplant health state A1 had a higher mean utility score (0.73) than A2 (0.63). Among the transplant year health states, B1 had the highest mean utility (0.62), followed by B2 (0.47), and B3 (0.39). Among the post-transplant health states, C1 had the highest mean utility score at 0.93, followed by C2 (0.75), and C3 (0.51). The order of mean utility scores among the chronic health states (the A and C states) and the path states (the B states) was identical to the order of preference in the introductory ranking task. There was only one participant whose order of utility scores did not match the ranking order.

There were no significant differences in utility by age, gender, or whether the respondents had dependents, which can sometimes affect utility valuations [52]. For six of the eight health states, there were no significant utility differences across the three geographic locations. However, the Bristol subgroup’s utility score for A2 was significantly lower than the utility of the London sample (0.52 vs. 0.67, *p* < 0.05). In addition, the Bristol utility score for C3 was significantly lower compared to both the London and Newcastle subgroups (0.36 vs. 0.57 and 0.54, respectively, *p* < 0.05).

With the exception of C1, most participants (> 87%) were willing to trade time to avoid living in any of the health states. For C1, only 81 participants (39.1%) were willing to trade time to avoid this state. Nearly all participants (> 95%) were willing to trade time to avoid A2, B2, B3, and C3. The great majority of participants rated each of the eight health states as better than dead (i.e., utility score > 0): A1, 99.0%; A2, 96.1%; C1, 99.5%; C2, 99.0%; C3, 92.8%; B1, 94.7%; B2, 92.3%; and B3, 91.3%.

### Utility differences

*T* tests revealed that all differences between health state utility pairs presented in Table 2 were statistically significant (*p* < 0.0001). Health states A1 and A2 had identical descriptions of TDT, except for the difference in type of iron chelation (oral vs. subcutaneous). The difference of 0.10 was statistically significant, indicating that there was a significant difference in preference between these two types of chelation. Both acute and chronic GvHD were associated with statistically significant disutilities (0.09 and 0.42, respectively). Gene addition therapy administered via autologous stem cell transplant was associated with a significantly greater utility than allogeneic transplant (utility difference = 0.15).

**Table 2:** Health state utility difference scores (*N* = 207)

Health state utility differences^a^	Mean	SD	95% CI
Utility difference: pre-transplant health states			
A1–A2: oral chelation vs. subcutaneous chelation	0.10	0.17	0.08 to 0.12
Utility differences: transplant health states			
B1–B2: gene addition therapy vs. allogeneic	0.15	0.17	0.13 to 0.17
B2–B3: allogeneic vs. allogeneic with acute GvHD	0.09	0.11	0.07 to 0.10
Utility differences: post-transplant health states			
C1–C2: transfusion independent vs. 60% reduction	0.18	0.21	0.15 to 0.21
C1–C3: transfusion independent vs. chronic GvHD	0.42	0.37	0.37 to 0.47
Utility differences: pre- vs post-transplant			
A1–C1: pre-transplant with oral chelation vs. post-transplant transfusion independent	− 0.21	0.21	− 0.24 to − 0.18
A2–C1: pre-transplant with subcutaneous chelation vs. post-transplant transfusion independent	− 0.31	0.29	− 0.35 to − 0.27
A1–C2: pre-transplant with oral chelation vs. post-transplant 60% reduction	− 0.03	0.07	− 0.04 to − 0.02
A1–C3: pre-transplant with oral chelation vs. post-transplant chronic GvHD	0.22	0.30	0.17 to 0.26
A2–C3: pre-transplant with subcutaneous chelation vs. post-transplant chronic GvHD	0.12	0.27	0.08 to 0.15

TTO scores are on a scale anchored with 0 representing dead and 1 representing full health

^a^*T* tests found that all differences between health state utility pairs presented in this table (e.g., A1 vs. A2, B1 vs. B2, …) were statistically significant (*p* < 0.0001)

## Discussion

In general, utilities followed expected patterns. The health state describing transfusion independence (C1), including associated benefits such as lack of symptoms and improved quality of life, had the highest utility score. The health states describing chronic TDT (health states A1 and A2) with the ongoing cycle of transfusion and chelation had substantially lower utilities. The difference in preference between health states describing transfusion independence and transfusion dependence highlights the considerable burden of this disease, as well as the benefits of potentially curative treatments such as gene addition therapy or stem cell transplant.

Utilities of health states differing in terms of treatment modality and treatment-related adverse events also followed logical patterns, with more difficult treatment processes associated with lower utility values. For example, TDT with subcutaneous chelation (A2) had a lower utility than an otherwise identical health state with oral chelation (A1). Chronic (C3) and acute GvHD (B3) were both associated with significant disutility. Furthermore, gene addition therapy (B1) had a significantly greater utility than allogeneic transplant (B2). The difference in preference between these two health states was likely related to the gene addition therapy having less impact on the immune system than allogeneic transplant. Specifically, health state B1 was likely perceived as advantageous because of the lack of immunosuppression therapy, fewer medications required after leaving the hospital, a shorter time of increased infection susceptibility in which exposure to other people must be limited, and a faster return to work.

A comparison to previously published results provides further support for the validity of the current findings. Two previous studies are relevant because they included TDT health state vignettes valued in TTO tasks by general population respondents [21, 23]. Neither study included health states similar to the B or C states in the current study, but both had health states describing TDT with either oral or subcutaneous chelation, similar to health states A1 and A2. As expected, the utility of health state A1 describing TDT with oral chelation (0.73) was lower than that of the parallel states in the other two studies (0.84 and 0.85). This difference is likely due to the more thorough description of the ongoing burden and risks of transfusion and chelation in the current study’s health state. The utility of health state A2 describing subcutaneous chelation (0.63) was similar to utilities of parallel states in the other two studies (0.66 and 0.61), likely because the subcutaneous chelation process was viewed as similarly aversive in all three studies. For the B and C health states, comparisons to previously published utilities are more challenging. For example, utilities associated with GvHD and transfusion reduction have been estimated in the context of other diseases such as myelogenous leukemia and MDS, but these may not be comparable to the TDT health states in the current study [27, 28, 30].

The utilities derived in this study may be useful in economic models examining and comparing the value of treatments for TDT. When modelers are using these values in a CUA, they should be aware of the difference between the chronic health states and the path states to ensure that the utilities are used correctly. The pre- and post-transplant health states (i.e., the A and C health states) were valued as chronic health states in a TTO task with a 10-year time horizon. Because these five health states did not change over time, the resulting utilities may be applied for any length of time in a CUA, consistent with the constant proportional trade-off assumption of the QALY model suggesting that the value of a health state is independent of the amount of time spent in that health state [53].

In contrast, the three transplant states (i.e., B1–B3) are path states each representing a 1-year time period in which the hypothetical patient proceeds through a series of health-related experiences, each of which lasts for a specified length of time. Therefore, the resulting utility values for these three path states cannot be considered independent of time, and they may only be used in a CUA to represent a 1-year period in which the transplant occurs. The advantage of this approach is that the utility represents the entire path, and respondents’ valuations are based on consideration of the full sequence of events as well as the time spent in each part of the path. The disadvantage is that it is not possible to identify the utility impact of individual parts of the path.

Although utilities derived in this study may be useful in economic modeling, limitations of the vignette-based methodology should be considered. Several HTA guidelines, including the guide issued by NICE, advocate for the use of utilities derived via generic preference-based measures such as the EQ-5D to maximize “consistency across appraisals” [34]. However, in situations when generic instruments are not sensitive to important aspects of disease or treatment, HTA guidelines allow for alternative utility assessment methodology. NICE specifies that models incorporating utilities estimated with other methodology may be acceptable when the EQ-5D is not “appropriate” [34].

For estimating utilities of TDT, generic instruments are likely to be inappropriate for two reasons. First, because TDT is a rare condition in much of the world including the US and UK [35, 54], it may not be feasible to recruit a large enough sample of patients representing each specific health state needed for modeling. Second, treatment process attributes are a key component differentiating among the relevant health states (e.g., oral vs. subcutaneous iron chelation; allogeneic stem cell transplant vs. gene addition therapy via autologous stem cell transplant). Generic instruments such as the EQ-5D were not designed to be sensitive to treatment process attributes. However, there is growing consensus that aspects of the treatment process are important to patients [31] and could therefore have an impact on health state preference. To estimate treatment process utilities, most researchers employ the vignette-based approach [31]. This approach is well-suited for assessing process utilities because the vignettes can be drafted to represent the typical experience of patients receiving treatment.

Still, an important limitation of vignette methods is that the resulting utility scores represent preferences for specific health states, rather than experience of an actual patient sample. The accuracy of the vignettes is inherently limited by the information on which they are based. For example, clinicians who were interviewed about the stem cell transplants said the patient experience is highly variable, and they were not able to estimate rates at which complications might occur. Therefore, risks of post-transplant complications (e.g., infection) are described, but the rates at which these risks occur could not be presented. Furthermore, the vignette approach cannot take into account the complexity of all possible events associated with TDT and its treatment, such as insertional oncogenesis, which is a potential risk associated with gene therapy or chemotherapy induced secondary malignancy with myeloablative conditioning. Therefore, the extent to which the reported utilities are comparable to values that might be derived from patients using generic instruments is not known. To mitigate this inherent limitation of the vignette approach, the TTO assessment methods were selected to maximize comparability to standardized instruments. For example, chronic health states were valued by UK general population participants in a TTO task with a 10-year time horizon, similar to methods used to derive the EQ-5D utility scoring tariffs [55].

Another limitation is that, although participants were from the UK general population, the participant recruitment strategy does not allow the sample to be considered nationally representative. Still, recruitment targets were set based on UK census data, and efforts were made to ensure that the sample was reasonably similar to the UK general population with regard to gender, age, and ethnic/racial background. For example, ethnic/racial background of the current sample (Table 1) is similar to rates in the UK census data, which has been reported as 87.2% White, 7.0% Asian, 3.0% Black, 2.0% mixed, and 0.9% other [56].

There may be additional limitations associated with health states B1–B3. For vignette-based utilities to be credible, it is essential that the content of the vignettes is clear and comprehensible to the respondents. These three health states were longer and more complex than typical vignettes used in utility valuations, and thus, they presented challenges for some participants. However, it seems that efforts to ensure participants understood the longer health states (as described in the Methods section) were generally effective because the resulting utilities followed logical patterns. The gene addition therapy as described in health state B1 was preferred over the allogeneic stem cell transplant in B2, indicating that most participants likely understood and considered the differences between these two longer health states. Nevertheless, it is possible that some participants may not have been able to consider all parts of the three longer health states, which could lead to some error in the resulting scores.

Another issue with health states B1–B3 is that they were valued on a 1-year time horizon because preference differences associated with the temporary transplant process would probably not be detected in the context of a longer time horizon. For TTO tasks, it has been shown that a reduction in remaining life expectancy does have an impact on responses [57], and this effect could have been amplified with the 1-year time horizon. Therefore, utilities for these three path states should be used and interpreted with caution.

Despite limitations, the current study represents an important step forward for economic modeling of treatment for TDT. The utilities derived in this study may be used in CUAs estimating the value of treatments that may eliminate or reduce the need for ongoing blood transfusions and iron chelation in these patients. In addition, differences among the health state utilities may be useful for distinguishing between approaches for iron chelation as well as between allogeneic stem cell transplant and the emerging gene addition therapy approach.

## Electronic supplementary material

Below is the link to the electronic supplementary material.

Supplementary material 1 (DOCX 97 kb)
