# Supplementary material for: Health state utilities associated with treatment for transfusion-dependent β-thalassemia
Source: Eur J Health Econ. 2019 Dec 11;21(3):397–407. doi: 10.1007/s10198-019-01136-0 (PMC7188724; doi:10.1007/s10198-019-01136-0)
Supplement: Supplementary file 1 — Supplementary material 1 (DOCX 97 kb) [file 10198_2019_1136_MOESM1_ESM.docx]

**APPENDIX A. HEALTH STATE TEXT**

For the interviews, the health state letters used throughout this article were changed so that respondents could rank the health states without being biased by the organizational structure implied by the original letters. For example, health state A1 was renamed as D, and A2 was renamed as M. The letters seen by the respondents are included in parentheses below.

Highlighted text indicates details that differ between similar health states.

**Health State A1: Pre-transplant β-Thalassemia with transfusion and oral chelation (D)**

**Disease**

- You have an **inherited blood disease** that makes it impossible for your body to make haemoglobin, which is the part of the blood that carries oxygen.
- This disease causes **severe anaemia**, which means there is not enough haemoglobin in your red blood cells to carry oxygen.
- Without regular blood transfusions, this disease would be fatal.

**Blood Transfusions**

- Treatment includes **blood transfusions**, which give you healthy red blood cells that are full of haemoglobin to correct your anaemia. The blood is from healthy blood donors.
- You receive these transfusions **every 3 to 4 weeks** at a hospital or transfusion unit.
- Approximately 2 days before each transfusion, you go to hospital for a blood test to make sure your blood is matched to the blood that is transfused.
- Blood transfusions are given through a tube inserted into a vein in your arm.
- Each transfusion lasts approximately **4 to 6 hours**.

**Removing Iron: Tablets**

- The blood transfusions cause a **build-up of iron** in your body. This iron can damage your heart, liver, hormone glands, and other vital organs.
- To reduce the amount of iron in your body, you must **take** **tablets daily**.
- This medication has **potential side effects** on your liver and kidney functioning, and may cause gastrointestinal upset or constipation. Therefore, your doctor will monitor you via blood tests and urine tests. Your doctor may adjust the dose as necessary. **Most patients can avoid these side effects with careful monitoring**.

**Symptoms and Impact**

- **In the days before your regular blood transfusions**:
  - You feel **tired and you may feel irritable**.
  - You may have **difficulty concentrating**.
  - You may not be as productive at **work or at school**.
  - Your ability to **exercise** may be limited.
  - You may feel an aching **pain** as if your bones hurt.
- You take **time off work or school** for appointments.
  - You go to the hospital for a **blood transfusion** every 3 to 4 weeks.
  - You go to the hospital for a **blood test** 2 days before each transfusion.
  - You have annual clinic appointments for liver and heart **scans** to check for iron build-up.
  - You have bone density **scans** every 18 months to check for osteoporosis (bone weakening).

**Health State A2: Pre-transplant β-Thalassemia with transfusion and subcutaneous chelation (M)**

**Disease**

- You have an **inherited blood disease** that makes it impossible for your body to make haemoglobin, which is the part of the blood that carries oxygen.
- This disease causes **severe anaemia**, which means there is not enough haemoglobin in your red blood cells to carry oxygen.
- Without regular blood transfusions, this disease would be fatal.

**Blood Transfusions**

- Treatment includes **blood transfusions**, which give you healthy red blood cells that are full of haemoglobin to correct your anaemia. The blood is from healthy blood donors.
- You receive these transfusions **every 3 to 4 weeks** at a hospital or transfusion unit.
- Approximately 2 days before each transfusion, you go to hospital for a blood test to make sure your blood is matched to the blood that is transfused.
- Blood transfusions are given through a tube inserted into a vein in your arm.
- Each transfusion lasts approximately **4 to 6 hours**.

**Removing Iron: Infusion**

- The blood transfusions cause a **build-up of iron** in your body. This iron can damage your heart, liver, hormone glands, and other vital organs.
- To reduce the amount of iron in your body, you must take medicine **using a small infusion pump about 5 days each week**.
- This medicine is **infused into your body through a needle** just under the surface of the skin of your abdomen. You can insert the needle by yourself.
- The infusion lasts **about 10 hours**. You can do this **during the day or night**.
  - **During the day**, you can carry the pump around in a small bag attached to your belt while performing most usual activities.
  - **At night**, the pump can be next to you in bed.
- This medication has **potential side effects** on your vision and hearing. Therefore, your doctor will monitor you via blood, urine, hearing, and vision tests. Your doctor may adjust the dose as necessary. **Most patients can avoid these side effects with careful monitoring**.
- You may experience pain, discomfort, and swelling at the infusion site.

**Symptoms and Impact**

- **In the days before your regular blood transfusions**:
  - You feel **tired and you may feel irritable**.
  - You may have **difficulty concentrating**.
  - You may not be as productive at **work or at school**.
  - Your ability to **exercise** may be limited.
  - You may feel an aching **pain** as if your bones hurt.
- You take **time off work or school** for appointments.
  - You go to the hospital for a **blood transfusion** every 3 to 4 weeks.
  - You go to the hospital for a **blood test** 2 days before each transfusion.
  - You have annual clinic appointments for liver and heart **scans** to check for iron build-up.
  - You have bone density **scans** every 18 months to check for osteoporosis (bone weakening).
  - You have **annual clinic appointments** to check your hearing and vision.

**Health State B1: Autologous Stem Cell Transplant (P)**

**Disease**

- To treat your blood disease, you receive a **stem cell transplant**.
- The potential **benefits** of transplant are that you may no longer need blood transfusions.
- The potential **risks** are that the transplant may not work, you may need additional transfusions, and you will be susceptible to bacterial and viral infections during recovery.

**Preparation for Transplant**

- You need a surgical procedure to insert an **intravenous line** into a large blood vessel. This line emerges from the skin on the side of your chest.
- You make **several visits** to the hospital for blood tests.
- You **stay in hospital for approximately 10 days** while preparing for the transplant. You undergo **chemotherapy** to eliminate your bone marrow and make space for the new stem cells. The chemotherapy is administered through the intravenous line over **several hours each day for several days**.
- You experience **nausea and tiredness**.

**Transplant**

- One or two days after chemotherapy is completed, you receive the **stem cell transplant** by infusion through the intravenous line. This infusion occurs on **one day, and it is not painful**.

**First Month after Transplant**

- You **stay in the hospital for approximately 1 month**. You stay in a **protective environment** because your immune system cannot fight germs.
- For the **first 2 weeks**, you experience mouth sores, sore throat, and diarrhoea. You are not able to eat or drink properly and you may need intravenous feeding. You may also have infections with fever.
- The chemotherapy can put you at risk of bleeding. To prevent this, you receive platelet infusions every few days. Platelets are a type of blood cell.
- You experience significant hair loss. Your hair starts to grow back after about 2 months.

**After Hospital**

- After a few months, your bone marrow produces enough red blood cells so that you **no longer need blood transfusions**.
- For **a month or two** after leaving the hospital, you try to **limit your exposure to other people** to avoid infections while your immune system is recovering from the chemotherapy.
- You take **a couple of medications** for a few months to help prevent infection during recovery.
- **Many people are re-admitted** to the hospital once or twice during the first 2 or 3 months after transplant because of complications such as infection.
- For about 3 months after leaving the hospital, you **visit the transplant unit** **once every week or two** for careful monitoring.
- You are able to **return to work or school approximately** **4 to 6 months** **after the transplant**.
- Starting about 6 months after the transplant, you begin repeating all the **vaccinations** you had as a child.

**Timeline**


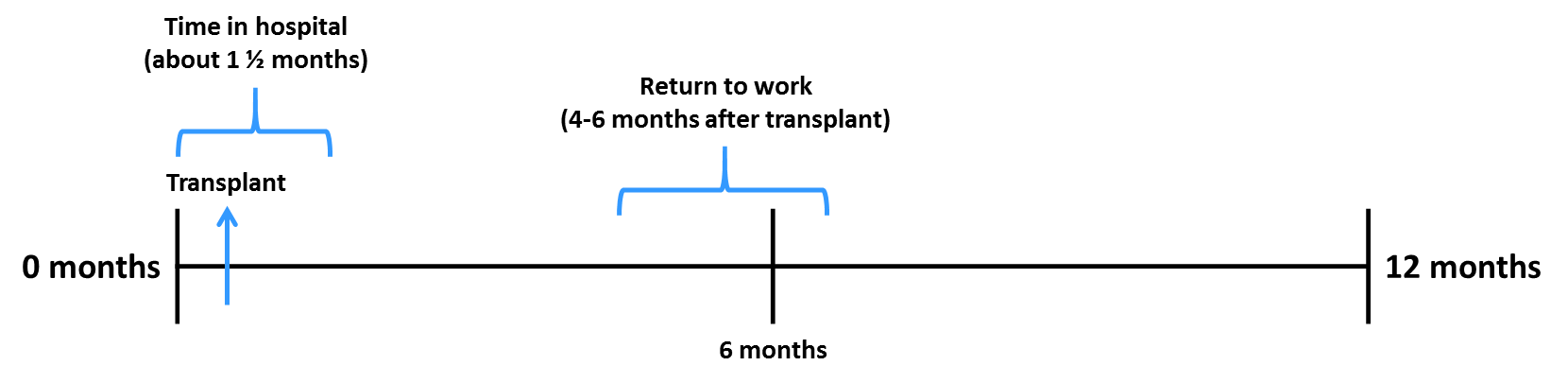


**Health State B2: Allogeneic Stem Cell Transplant (S)**

**Disease**

- To treat your blood disease, you receive a **stem cell transplant**.
- The potential **benefits** of transplant are that you may no longer need blood transfusions.
- The potential **risks** are that the transplant may not work, you may need additional transfusions, and you will be susceptible to bacterial and viral infections during recovery.

**Preparation for Transplant**

- You need a surgical procedure to insert an **intravenous line** into a large blood vessel. This line emerges from the skin on the side of your chest.
- You make **several visits** to the hospital for blood tests.
- You **stay in hospital for approximately 10 days** while preparing for the transplant. You undergo **chemotherapy** to eliminate your bone marrow and make space for the new stem cells. The chemotherapy is administered through the intravenous line over **several hours each day for several days**.
- You experience **nausea and tiredness**.
- You receive **medication to suppress your immune system**. This prevents your body from attacking the transplanted stem cells so that new bone marrow can grow. During this time, **your immune system is seriously compromised**, and you are at risk of infection.

**Transplant**

- One or two days after chemotherapy is completed, you receive the **stem cell transplant** by infusion through the intravenous line. This infusion occurs on **one day, and it is not painful**.

**First Month after Transplant**

- You **stay in the hospital for approximately 1 month**. You stay in a **protective environment** because your immune system cannot fight germs.
- For the **first 2 weeks**, you experience mouth sores, sore throat, and diarrhoea. You are not able to eat or drink properly and you may need intravenous feeding. You may also have infections with fever.
- The chemotherapy can put you at risk of bleeding. To prevent this, you receive platelet infusions every few days. Platelets are a type of blood cell.
- You experience significant hair loss. Your hair starts to grow back after about 2 months.

**After Hospital**

- After a few months, your bone marrow produces enough red blood cells so that you **no longer need blood transfusions**.
- You continue to take **medication to suppress your immune system** for about **6 months** after the transplant. During this time, you try to **limit your exposure to other people** to avoid infections while your immune system is suppressed.
- You take **several (up to 10) medications daily** to help prevent infection and support your body during recovery. Some of these medications can make you feel **nauseous**.
- **Many people are re-admitted** to the hospital once or twice during the first 2 or 3 months after transplant because of complications such as infection.
- For about 3 months after leaving the hospital, you **visit the transplant** **unit** **once every week or two** for careful monitoring.
- You are able to **return to work or school approximately 9 to 12 months after the transplant**.
- Starting about 6 months after the transplant, you begin repeating all the **vaccinations** you had as a child.

**Timeline**


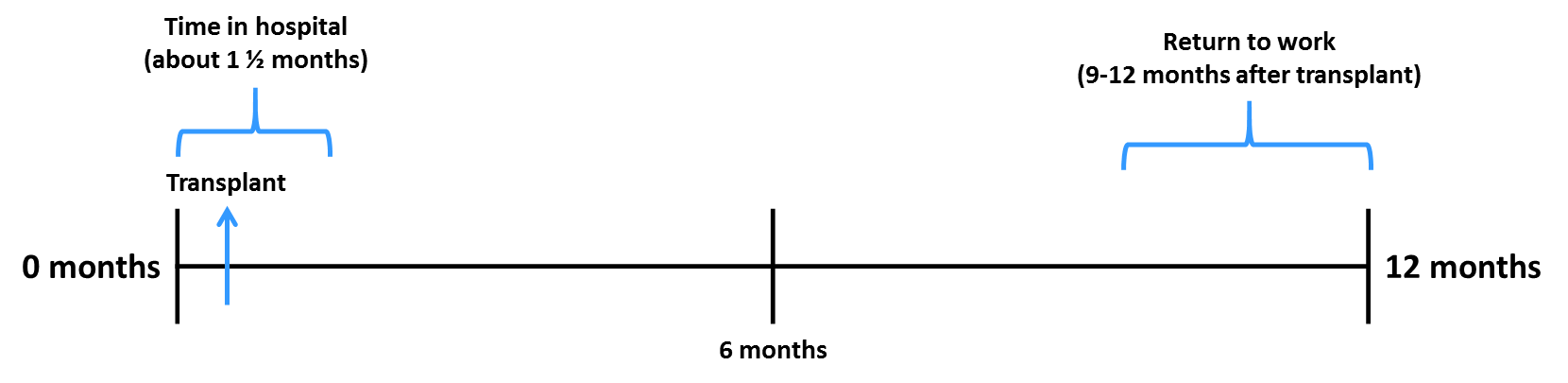


**Health State B3: Allogeneic Stem Cell Transplant with Acute GvHD (G)**

**Disease**

- To treat your blood disease, you receive a **stem cell transplant**.
- The potential **benefits** of transplant are that you may no longer need blood transfusions.
- The potential **risks** are that the transplant may not work, you may need additional transfusions, and you will be susceptible to bacterial and viral infections during recovery.

**Preparation for Transplant**

- You need a surgical procedure to insert an **intravenous line** into a large blood vessel. This line emerges from the skin on the side of your chest.
- You make **several visits** to the hospital for blood tests.
- You **stay in the hospital for approximately 10 days** while preparing for the transplant. You undergo **chemotherapy** to eliminate your bone marrow and make space for the new stem cells. The chemotherapy is administered through the intravenous line over **several hours each day for several days**.
- You experience **nausea and tiredness**.
- You receive **medication to suppress your immune system**. This prevents your body from attacking the transplanted stem cells so that new bone marrow can grow. During this time, **your immune system is seriously compromised**, and you are at risk of infection.

**Transplant**

- One or two days after chemotherapy is completed, you receive the **stem cell transplant** by infusion through the intravenous line. This infusion occurs on **one day, and it is not painful**.

**First Month after Transplant**

- You **stay in the hospital for approximately 1 month**. You stay in a **protective environment** because your immune system cannot fight germs.
- For the **first 2 weeks**, you experience mouth sores, sore throat, and diarrhoea. You are not able to eat or drink properly and you may need intravenous feeding. You may also have infections with fever.
- The chemotherapy can put you at risk of bleeding. To prevent this, you receive platelet infusions every few days. Platelets are a type of blood cell.
- You experience significant hair loss. Your hair starts to grow back after about 2 months.

**Complication**

- About 3 weeks after the transplant, **the transplanted cells begin to attack the cells in your body**. This keeps you **in the hospital for an extra 2 weeks**.
- This causes a skin rash, sickness, weight loss, loss of appetite, severe diarrhoea, and severe abdominal pain.
- You are treated with intravenous steroids for a few days, followed by oral steroids for about 2 months. This condition resolves in a month or two.

**After Hospital**

- After a few months, your bone marrow produces enough red blood cells so that you **no longer need blood transfusions**.
- You continue to take **medication to suppress your immune system** for about **6 months** after the transplant. During this time, you try to **limit your exposure to other people** to avoid infections while your immune system is suppressed.
- You take **several (up to 10) medications daily** to help prevent infection and support your body during recovery. Some of these medications can make you feel **nauseous**.
- **Many people are re-admitted** to the hospital once or twice during the first 2 or 3 months after transplant because of complications such as infection.
- For about 3 months after leaving the hospital, you **visit the transplant unit** **once every week or two** for careful monitoring.
- You are able to **return to work or school approximately 9 to 12 months after the transplant**.
- Starting about 6 months after the transplant, you begin repeating all the **vaccinations** you had as a child.

**Timeline**


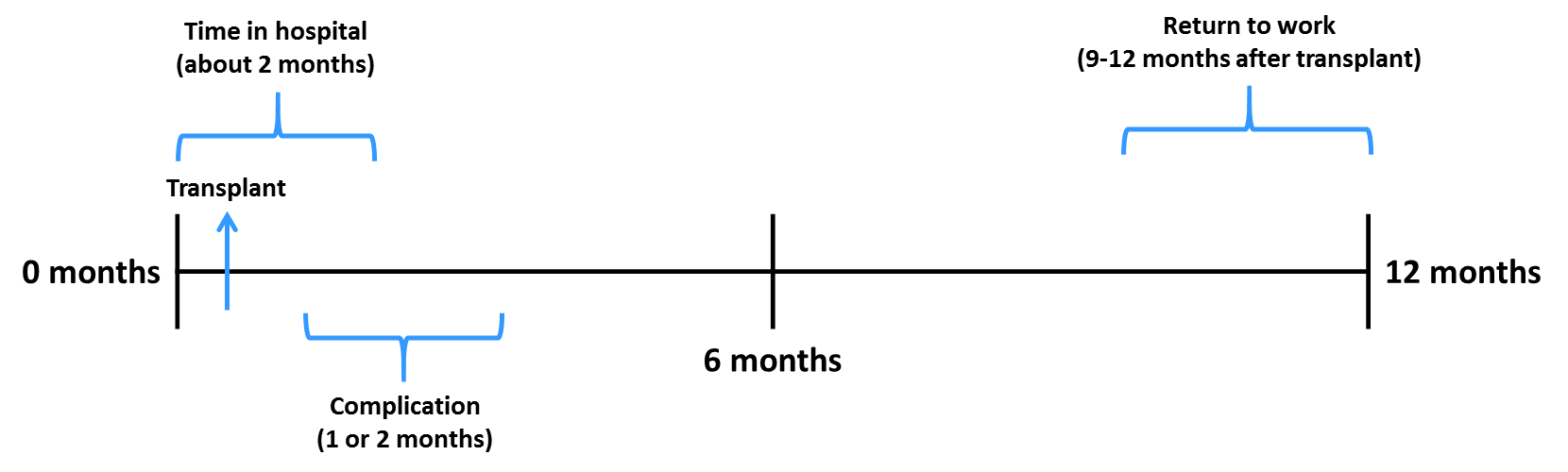


**Health State C1: Post-Transplant, Transfusion Independent (U)**

**Disease Status**

- **You received a stem cell transplant in the past.**
- Your bone marrow now produces normal red blood cells full of haemoglobin.
- You **do not** have anaemia.

**Blood Transfusions**

- You no longer need to receive blood transfusions.

**Removing Iron**

- You **do not** require treatment to remove excess iron from your body.

**Symptoms and Impact**

- You **do not** have any symptoms of your previous blood condition.
- Your history of the blood disease does not affect your current ability to work or engage in your usual activities.
- You have **annual follow-up visits** with your doctor to monitor the long-term effects of chemotherapy and stem cell transplant.

**Health State C2: Post-Transplant, 60% Transfusion Reduction in Terms of Volume (Y)**

**Disease Status**

- **You received a stem cell transplant in the past**.
- Your bone marrow now produces **some** normal red blood cells full of haemoglobin, **but you still have anaemia**.
- Without regular blood transfusions, this disease would be fatal. This condition requires ongoing treatment and hospital visits.

**Blood Transfusions**

- Treatment includes **blood transfusions**, which give you healthy red blood cells that are full of haemoglobin to correct your anaemia. The blood is from healthy blood donors.
- You receive these transfusions **every 6 to 8 weeks** at a hospital or transfusion unit.
- Approximately 2 days before each transfusion, you go to hospital for a blood test to make sure your blood is matched to the blood that is transfused.
- Blood transfusions are given through a tube inserted into a vein in your arm.
- Each transfusion lasts approximately **4 to 6 hours**.

**Removing Iron: Tablets**

- The blood transfusions cause a **build-up of iron** in your body. This iron can damage your heart, liver, hormone glands, and other vital organs.
- To reduce the amount of iron in your body, you must **take tablets daily**.
- This medication has **potential side effects** on your liver and kidney functioning, and may cause gastrointestinal upset or constipation. Therefore, your doctor will monitor you via blood tests and urine tests. Your doctor may adjust the dose as necessary. **Most patients can avoid these side effects with careful monitoring**.

**Symptoms and Impact**

- **In the days before your regular blood transfusions**:
  - You feel **tired and you may feel irritable**.
  - You may have **difficulty concentrating**.
  - You may not be as productive at **work or at school**.
  - Your ability to **exercise** may be limited.
  - You may feel an aching **pain** as if your bones hurt.
- You take **time off work or school** for appointments.
  - You go to the hospital for a **blood transfusion** every 6 to 8 weeks.
  - You go to the hospital for a **blood test** 2 days before each transfusion.
  - You have annual clinic appointments for liver and heart **scans** to check for iron build-up. You have bone density **scans** every 18 months to check for osteoporosis (bone weakening).

**Health State C3: Post-Transplant, Chronic GvHD (R)**

**Disease Status**

- **You received a stem cell transplant in the past**.
- Your bone marrow now produces normal red blood cells full of haemoglobin.
- You **do not** have anaemia.

**Blood Transfusions**

- You no longer need to receive blood transfusions.

**Removing Iron**

- You **do not** require treatment to remove excess iron from your body.

**Symptoms and Impact**

- **The transplanted cells attack the cells in your body**. This causes a range of symptoms:
- You experience a **skin rash** with skin thickening and tightening. The skin rash affects your appearance.
- You have **chronic diarrhoea**, unintentional weight loss, and abdominal pain.
- You experience **stiffening of your joints**. This is painful and persistent, and it causes you to limit your physical activity.
- You have **dry mouth and dry eyes**. This can be uncomfortable.
- You are **prone to infection**, including respiratory and fungal infections.
- Your symptoms interfere with your ability to be productive at work and school.
- You are not very active because you **often feel sick**.
- You take time off work or school for appointments associated with treatment.

**Treatment**

- You take **oral medication daily to suppress your immune system**. This may include oral steroid medication.
- You also receive intravenous infusions once **every 6 weeks** to help strengthen your immune system.
